# The national dental undergraduate clinical skills competition known as the Guanghua Cup: an innovative and effective program that promoted undergraduate dental education in China

**DOI:** 10.1186/s12909-021-02841-2

**Published:** 2021-07-27

**Authors:** Xiaolan Li, Yanbo Shan, Yangjingwen Liu, Yingwen Lin, Lin Li, Zhengmei Lin, Shuheng Huang, Yun Hong

**Affiliations:** Hospital of Stomatology, Guanghua School of Stomatology, Guangdong Provincial Key Laboratory of Stomatology, Sun Yat-Sen University, 56 Lingyuan Road West, Guangzhou, 510055 Guangdong China

**Keywords:** Competition, Clinical skills, Undergraduate dental education, Teaching quality

## Abstract

**Background:**

The National Dental Undergraduate Clinical Skills Competition known as the Guanghua Cup was held in Guangzhou, China, for three consecutive years from 2017 to 2019 to promote the clinical teaching of undergraduate dental education and to enhance communication among different universities. The present study aimed to introduce the organization, procedures, and consequences of the competition, in addition to analyzing the influences of competition on the reform of undergraduate dental education.

**Methods:**

By analyzing the descriptive statistics of the Guanghua Cup, the competitions’ organization, the participating students’ performances, and the outcomes of competitions were analyzed. After distributing questionnaires to all participants of the 2nd and 3rd Cups, their attitudes towards the competition and their evaluation of the role of the competitions in promoting undergraduate dental education were analyzed.

**Results:**

A total of 24 schools participated in the 3 competition years. The contents of the competitions covered cariology, endodontics, periodontology, prosthodontics, oral and maxillofacial surgery, dental anatomy, and first aid (e.g., operative skills and theoretical knowledge). Compared with those of the 2nd Cup, the mean scores of the operative skills significantly improved in the stations related to periodontology, prosthodontics, and dental anatomy (*p* < 0.05) in the 3rd Cup. In addition, 338 valid questionnaires were collected, for a response rate of 87.79 %. Overall, the participants spoke highly of the Guanghua Cup. Based on their self-perception and self-evaluation, the majority of interviewees agreed that the competition helped develop collegiality and teamwork among the participating students, improved the students’ clinical skills and promoted the improvement of teaching resources (e.g., purchasing and updating equipment, models or experimental materials).

**Conclusions:**

The competition enjoyed the widest coverage since it involved dental schools from all of the different geographical regions of China. Dental students could exhibit their clinical skills in a competitive environment and develop collegiality and teamwork. Future competitions should be optimized through their organization and contents. The education quality of the participating schools affected by such competition should be investigated in a more objective and comparable way.

## Background

In recent decades, dental education has significantly progressed in China. At present, there are 114 universities or dental schools that enroll undergraduate students, and more than 10,000 dental undergraduates graduate annually in China.

The dental education system in China follows the higher education system of the former Soviet Union, which positions dental education in the medical branch of study. According to this system, a 5-year undergraduate education includes three stages: basic medical education, clinical medical education, and dental education [1]. Therefore, in China, it is called stomatology rather than dentistry. Although the curriculum programs are not the same among different universities, undergraduate students generally start their dentistry studies in their 3rd year. In the following 2–3 years, they take dental courses, engage in laboratory (preclinical) training, and do a one-year rotation of clinical practice. With the large population in China, Chinese dental students can interact with a large number of patients in the clinical practice stage and gain rich clinical experience. However, compared with Western countries [2, 3] and other Asian countries [4], the total time spent on preclinical training at the undergraduate level is limited. Therefore, it is highly essential to obtain sufficient high-level dental preclinical training to not only eliminate the gap between the preclinical stage and clinical practice but also to improve the safety of patients and ensure the quality of dental education.

To enhance the quality of teaching and regulate teaching behaviors among different dental schools, The Society of Dental Education, Chinese Stomatological Association, has formulated the Standards of Clinical Practice for Chinese Undergraduate Students Majoring in Stomatology [5, 6]. In 2018, the China Higher Teaching Guidance Committee of the Ministry of Education issued “The Standards for Undergraduate Students Majoring in Stomatology in China”. These standards are not only reliable references for dental teaching but also significant criteria for quality evaluation.

Through national conferences, teaching and research forums, and exchange visits, the standardization and quality of dental teaching have been significantly improved across China. At the same time, dental skills competitions organized by domestic dental schools and institutions have emerged and become increasingly popular as they support the education and training of dental students. For instance, the West China School of Stomatology, Sichuan University (Chengdu, China) held the International Dental Undergraduate Competition from 2014 to 2019. The Guanghua School of Stomatology, Sun Yat-sen University (Guangzhou, China) held the National Dental Undergraduate Clinical Skills Competition known as the Guanghua Cup from 2017 to 2019. The Air Force Military Medical University (Xi’an, China) hosted the National Dental Undergraduate Skills Competition known as the Silk-Road Cup in 2019 and 2020. In addition, Shanghai Jiao  Tong University (Shanghai, China), Xi’an Jiaotong University (Xi’an, China), etc. have held similar dental undergraduate clinical skills competitions that achieved promising results and promoted the development of regional dental teaching.

The National Dental Undergraduate Clinical Skills Competition known as the Guanghua Cup (formerly known as the South China Cup) is an annual event that first ran from 2017 to 2019 [7]. It originated from the Annual Dental Skills Competition for Students of the Guanghua School of Stomatology, Sun Yat-sen University. The Guanghua Cup was established to assist dental students with an opportunity to demonstrate their clinical skills and was one of the largest national competitions. The present study aimed to introduce the organization and procedures of the competition, analyze the influence of the competition on the reform of undergraduate dental education, and provide new ideas for future dental skills competitions.

## Methods

### 1. Organization

The competitions were hosted by the Guanghua School of Stomatology, Sun Yat-sen University. Each year, 1–4 of the top dental schools of each geographic area in China were invited to participate in the competition. The competitions were held in the preclinical training center of the Guanghua School. The organizer introduced the contents and rules of the competition via websites, emails, and WeChat in advance.

### 2. Participants

Participants were dental students in their fifth-year of clinical practice. A team of four students from each school was selected through a school-level competition, and each team represented its school. The prizes were ultimately awarded to eligible students and schools.

### 3. Competition design

Dental experts from participating schools designed the competition projects and developed the scoring standards. The organizers of the competition announced the competition items, rules, and scoring systems in advance. The competition mainly evaluated the professional theoretical knowledge, clinical operative skills, and first aid ability of the participants.

### 4. Competition rules

**Fig. 1:**
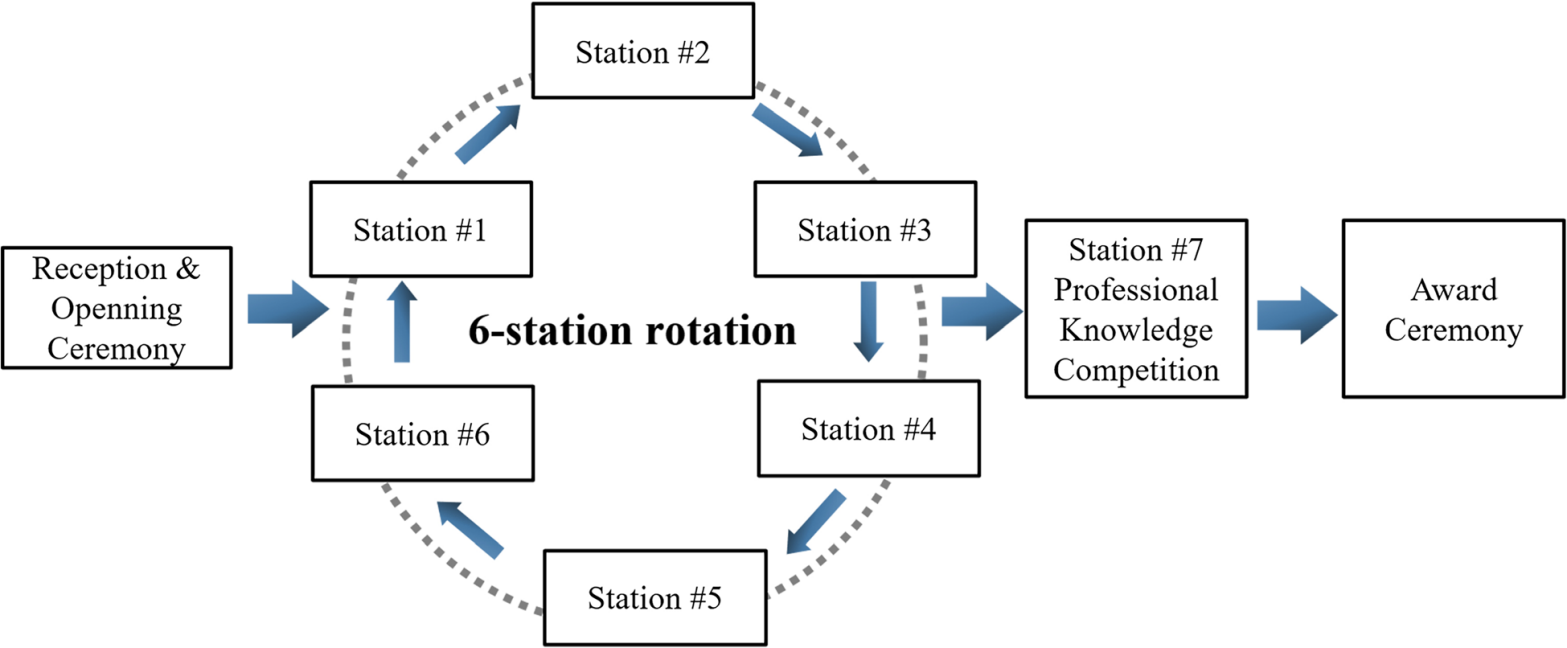
Flowchart of the National Dental Undergraduate Clinical Skills Competition (the Guanghua Cup)

Based on the successful holding of the first competition, the event was organized in each of the following two years. The rules of the 2nd and 3rd competitions were similar, with both comprising two sections with a total of 7 stations. Generally, the first part of the competition focused on operative skills (stations #1 to #6), which was followed by the professional knowledge competition (station #7) in the second part. To ensure the fairness of the game, the first 6 stations were run simultaneously, and each team drew lots to determine its starting station. That is, four teams started their skill competitions simultaneously at each station. Each team stayed at each station for 25 min. The participants completed the test individually or in pairs according to the competition rules and were subsequently scored. After that, the teams turned to the next station according to the directional arrows, until they passed through all 6 stations. All the teams performed the professional knowledge competition together at a conference hall. The competition was conducted in a multiple-choice format. After a question was asked, the first team that pressed down the answer light was allowed to respond. Points were added for correct answers. In contrast, points were deducted for incorrect responses. Finally, awards were conferred to the four teams with the top four point totals from each station. At the award ceremony, representatives of the referees commented on the roles of the participating students, pointed out their shortcomings, and summarized their operative skills (Fig. 1).

### 5. Contents of competitions

The contents of the competitions included the seven clinical operative skills of cariology, endodontics, periodontology, prosthodontics, oral and maxillofacial surgery, dental anatomy, and first aid, as well as clinical and theoretical knowledge that must be mastered by dental undergraduate students. A standardized patient (SP), simulated human model, phantom head (dentition with extracted teeth and plastic teeth), wax block used for carving or molding, and skin model of pig trotters were utilized. Station #1 included G.V. Black caries classification class II cavity preparation, access cavity preparation, and rubber dam isolation; station #2 consisted of subgingival scaling and periodontal suture; station #3 contained head bandaging and surgical suture; station #4 consisted of dental impressions, ceramic crowns and veneer preparation; station #5 included tooth carving and tooth molding; and station #6 contained cardiopulmonary resuscitation (CPR) and blood pressure (BP) measurement. The contents of the knowledge competition at station #7 covered the etiology, symptoms and signs, diagnosis, and treatment principles of all oral diseases. The test items of the competition covered 12 clinical dental skills and scoring standards (Table 1). For cavity preparation, 80 % of the score originated from the referees’ evaluation and 20 % originated from the automatic score of the Digital Tooth Carving Evaluation System (Fair Grader 2000; Nissin Dental Products Inc., Kyoto, Japan) [8]. For ceramic crown or ceramic veneer preparation, 80 % of the score was from the referees’ evaluation and 20 % was from the Real-time Dental Training and Evaluation System (RDTES, Ai Zhixing, Dcarer, China) [9].

**Table 1:** Details of the previous 3 annual competitions

Station	Subject	Content of assessed skills	Object	Competition year		
				2017	2018	2019
1	Operative Dentistry and Endodontics	G. V. Black class II cavity preparation	Phantom head with plastic teeth dentition	*	*	*
		Access cavity preparation	Phantom head with extracted teeth dentition	*	*	*
		Rubber dam isolation	Phantom head with plastic teeth dentition	*	*	*
2	Periodontology	Subgingival scaling	Silica gel periodontic teeth mould with plastic teeth		*	*
		Periodontal suture	Silica gel periodontic teeth mould with plastic teeth		*	*
3	Oral and Maxillofacial Surgery	Head bandaging	One of the team members	*	*	*
		Surgical suture	Pig's trotters	*	*	*
4	Prosthodontics	Dental impression	Standardized patient (SP)	*	*	*
		Ceramic crown preparation	Phantom head with plastic teeth dentition	*	*	*
		Porcelain veneer preparation	Phantom head with plastic teeth dentition	*	*	*
5	Dental Anatomy	Tooth carving	Wax block used for carving		*	*
		Tooth crown molding	Wax and plastic teeth used for crown molding		*	*
6	First Aid	Cardiopulmonary resuscitation (CPR)	Standardized simulated human model	*	*	*
		Blood pressure (BP) measurement	One of the team members	*	*	*
7	Knowledge competition	Knowledge covered the etiology, symptoms and signs, diagnosis and treatment principles of all oral diseases.	Not Applicable	*	*	*

* Competition item

### 6. Referees

Each participating institution nominated an expert who was a senior clinician with expertise in the corresponding station for the judgment of the competition. Station #6, i.e., CPR and BP, was judged by clinical experts. There were 2–6 referees at each station. The referees were responsible for scoring each team’s performance at each station, and the teams’ scores were averaged. A training meeting for the referees was held before the competition to properly train the referees. Through thus training, the referees became familiar with the competition rules, procedures, evaluation forms, and scoring standards. In addition, the referees were reminded about the permitted actions. On the day before the competition, the referees arrived at the competition field. They were familiarized with the equipment with the assistance of a station master (a station master is a person who is introduced by the organizer).

### 7. Analysis of effectiveness

Using the descriptive and data analysis of the 2nd and 3rd Guanghua Cups, the organization, participants’ performance, influence, and guidance effect of the competitions were analyzed. By distributing questionnaires powered by www.wjx.cn to all the participants, referees, team leaders, and volunteers, their perceptions and attitudes towards the competition were collected and analyzed. The questionnaires comprised basic information about the respondents, their evaluation of and satisfaction with the competition, benefits for individuals and colleges, and suggestions for future competitions. In addition, precompetition training information was collected from all the schools that participated in the 2nd and 3rd Guanghua Cups.

### 8. Statistical analysis

Data were statistically analyzed by using SPSS 21.0 software (IBM, Armonk, NY, USA). A *t*-test was used to compare the mean scores of the participants’ clinical operative skills at each station in the 2nd and 3rd Cups. One-way analysis of variance (ANOVA) or the Kruskal-Wallis test were utilized to compare differences in the evaluation of the influence of competition on undergraduate dental education among different groups. Fisher’s exact test was used to compare the selection differences among different groups on the possibility of personal ability improvement and school teaching influenced by the Guanghua Cup, as well as suggestions for other clinical skills provided for future competitions. Finally, *p* < 0.05 was considered statistically significant.

## Results

### 1. Participation in the competition

Since the competition was first held in 2017, the number of participating schools gradually increased every year. A total of 24 schools with 228 undergraduate dental students participating in the competition and 57 teachers serving as referees participated in the competition between 2017 and 2019. Additionally, special guests (deans or education administrators of the participating schools) and volunteer staff (junior dental students from the Guanghua School of Stomatology) joined the events. The competition involved 12 (1st ), 21 (2nd ), and 22 (3rd ) top-grade dental schools, which covered all the geographical regions in China. Moreover, 2 overseas schools were invited to participate in the 3rd year of competition in 2019 (Table 2).

**Table 2:** The schools that participated in the previous 3 annual competitions

Geographical region^a^	University (School) ^a^	2017	2018	2019
Eastern China	Nanjing Medical University (School of Stomatology)		*	*
	Shanghai Jiao Tong University (School of Stomatology)	*	*	*
	Shandong University (School of Stomatology)		*	*
	Tongji University (School of Stomatology)		*	*
	Zhejiang University (School of Stomatology)	*	*	*
Middle China	Wuhan University (School of Stomatology)	*	*	*
Northern China	Capital University of Medical Sciences(School of Stomatology)	*	*	*
	Tianjin Medical University (School of Stomatology)		*	*
	Peking University (School of Stomatology)	*	*	*
Northern East	Dalian Medical University (School of Stomatology)		*	*
	Harbin Medical University (School of Stomatology)		*	*
	Jilin University (School of Stomatology)	*	*	*
Northern West	Air Force Medical University (School of Stomatology)	*	*	*
	Xi’an Jiaotong University (School of Stomatology)	*	*	*
	Xinjiang Medical University (School of Stomatology)			*
Southern China	Guangxi Medical University (School of Stomatology)		*	*
	Guangzhou Medical University (School of Stomatology)		*	*
	Southern Medical University (School of Stomatology)	*	*	*
	Sun Yat-sen University (Guanghua School of Stomatology)	*	*	*
Southern West	Chongqing Medical University (School of Stomatology)		*	*
	Sichuan University (West China School of Stomatology)	*	*	*
Overseas	Khon Kaen University (Faculty of Dentistry), Thailand			*
	Temple University (Kornberg School of Dentistry), USA			*
Total number of schools		12	21	24

^a^ Sort by alphabetical order.

* Participated.

### 2. Training before the competition

Eighteen participating schools (response rate of 75.00 %) offered precompetition training information. They organized the training for all the students at 3 to 5 weeks (mean ± sd = 3.81 ± 0.69 weeks) before the competition. After the competition team selection, the schools focused on the training for the participating team. 100 % of the schools used weekend days (mean ± sd = 3.31 ± 1.02 days, 8 h/day) and weekday nights (mean ± sd = 4.03 ± 0.98 nights, 2–3 h/night) for student training before the competition. A total of 83.33 % of the schools used weekdays for training before the competition (mean ± sd = 1.22 ± 0.71 days, 8 h/day). In general, the total training time was 44.76 ± 8.09 h per participating student.

### 3. Participants’ performance in the operative skills in the Guanghua Cups

Table 3 showed the mean scores of the operative skills of all the participating schools from each station in the 2nd and 3rd Guanghua Cups. Compared with the 2nd Cup, the mean scores of subgingival scaling and periodontal suture (station #2), dental impression, ceramic crown and porcelain veneer preparation (station #4), tooth carving, and modeling (station #5) were improved in the 3rd Cup (*p* < 0.05).

**Table 3:** Average operating skills scores of all the participating schools at each station of the 2nd and 3rd Cups

Station	Operating skills	Average score (Mean ± sd)		*t*-test *(p* value)
		3rd	2nd	
1	G. V. Black class II cavity preparation	72.49 ± 5.12	73.03 ± 7.39	-0.281 (0.780)
	Access cavity preparation			
	Rubber dam isolation			
2	Subgingival scaling	89.28 ± 1.97	86.64 ± 2.40	3.997 (0.000)*
	Periodontal suture			
3	Head bandaging	79.18 ± 5.35	79.10 ± 7.30	0.041 (0.967)
	Surgical suture			
4	Dental impression	81.33 ± 3.59	78.81 ± 3.65	2.3293 (0.025)*
	Ceramic crown preparation			
	Porcelain veneer preparation			
5	Tooth carving	79.19 ± 4.93	60.22 ± 5.08	12.661 (0.000)*
	Tooth molding			
6	Cardiopulmonary resuscitation (CPR)	83.56 ± 5.21	86.47 ± 5.09	-1.890 (0.066)
	Blood pressure (BP) measurement			

A* t*-test compared the scores between the 2nd and 3rd Cups. *(*p* < 0.05)

### 4. Outputs of the Questionnaire

#### 4.1 Respondents’ basic information

After distributing the questionnaire to those who joined the 2nd and 3rd Guanghua Cups, including referees, team leaders, participating students, and volunteers (junior dental students), 338 valid questionnaires were collected from respondents, specifically, 32 referees, 30 team leaders, 166 students, and 110 volunteers, with response rates of 71.11 %, 75.00 %, 96.51 %, and 85.94 %, respectively. Except for a slightly higher number of female referees compared with male referees, the male:female ratio of the other three identities was close to 3:7. In addition, 20 % of the interviewees participated in the first competition. Approximately half of the team leaders attended similar competitions held either in China or abroad, while the majority of the referees and students had never experienced such attendance (approximately 70 %). When they received the questionnaire survey, the students and volunteers had graduated. The majority of the students and volunteers were postgraduate students (79.00 %), followed by residents (13.00 %). In terms of professional titles, all the referees were professors or associate professors, and more than half of the team leaders (66.67 %) were lecturers.

#### 4.2 Evaluation of and satisfaction with the competition

Referees, team leaders, and participating students were asked to indicate their level of agreement on a 10-point scale, ranging from 1 (strongly disagree) to 10 (strongly agree) as labeled, for each statement. The questions were focused on the effects and influence of the competition, including enhancing both clinical skills and theoretical knowledge, developing collegiality and teamwork, expanding domestic or international communication, promoting the improvement of teaching resources, motivating faculty training and encouragement, and benefitting all students (not limited to the participating students) (Table 4 Q1-12).

**Table 4:** Results of the questionnaire on the effects of the competition on undergraduate dental education

Questions	Mean ± sd				*p* value
	referees	team leaders	participating students	total interviewees	
Q1. The competition encourages colleges to attach more attention to the training of students’ clinical skills.	7.63 ± 2.03*	9.03 ± 1.30	9.02 ± 1.09	8.82 ± 1.37	0.000
Q2. The competition encourages colleges to attach more attention to the teaching of students’ theoretical knowledge.	6.91 ± 2.15*	8.33 ± 1.67	8.17 ± 1.65	8.01 ± 1.78	0.004
Q3. The competition improves the participating students’ clinical skills.	8.44 ± 1.64*	9.17 ± 1.44	9.24 ± 1.00	9.12 ± 1.20	0.016
Q4. The competition improves the participating students’ theoretical knowledge.	7.69 ± 2.09**	8.20 ± 1.35	8.60 ± 1.53**	8.42 ± 1.62	0.019
Q5. The competition helps develop collegiality and teamwork among participating students.	8.94 ± 1.32	9.47 ± 0.97	9.06 ± 1.30	9.10 ± 1.27	0.132
Q6. The competition helps expand domestic or international communication and broaden mind among participants.	8.16 ± 2.05	9.17 ± 1.29	8.68 ± 1.57	8.67 ± 1.62	0.070
Q7. The competition promotes the improvement of teaching resources (e.g., purchasing and updating equipment, models or experimental materials, etc.)	8.19 ± 1.40*	8.93 ± 1.26	8.78 ± 1.52	8.72 ± 1.48	0.023
Q8. The competition promotes the improvement of faculty training and encouragement (e.g., providing teachers with training opportunities, increasing teaching subsidies, or offering teaching rewards).	7.31 ± 2.18	7.70 ± 1.68	N/A	7.50 ± 1.95	0.438
Q9. During the event, the one-to-one liaison (junior dental student volunteers) arranged by the organizer provides professional and timely solutions to competition-related issues.	9.06 ± 1.22	9.50 ± 0.94	9.28 ± 1.28	9.28 ± 1.23	0.177
Q10. Precompetition training or selection improves the clinical skills of all students (not limited to the participating students).	6.78 ± 2.70	7.60 ± 1.65	N/A	7.18 ± 2.27	0.158
Q11. Precompetition training or selection improves the theoretical knowledge of all students (not limited to the participating students).	6.66 ± 2.36	7.40 ± 1.92	N/A	7.02 ± 2.18	0.181
Q12. Overall evaluation of the Guanghua Cup (formerly known as the South China Cup)	8.69 ± 1.38*	9.37 ± 0.93	8.89 ± 1.03	8.93 ± 1.10	0.032

* Represent statistically significant difference between the respondent group and the other two ones (*p* < 0.05).

** Represent statistically significant difference between the two respondent groups (*p* < 0.05).

*N/A* Not Applicable

The three items with the highest average scores (greater than 9 points) are presented in Table 4. During the competition, the interviewees reported excellent satisfaction with the one-to-one liaison (Q9), followed by the other two most agreeable issues, namely, improving the participating students’ clinical skills and collegiality and teamwork (Q3 and 5). In contrast, the three items with the lowest average score (less than 7.5 points) suggested that the interviewees thought that the competition played an insignificant positive role in promoting faculty training and encouragement (Q8). Although precompetition training and/or the selection of the students happened at all the schools, improvement in all students’ clinical skills and theoretical knowledge was not agreed upon highly by the referees and the team leaders (Q10 and 11). Although all the interviewees agreed with each topic (greater than 7 points), there were significant differences in the degree of identification. Generally, team leaders and participating students were more inclined to agree (higher scores). Overall, all interviewees spoke highly of the Guanghua Cup (Q12).

#### 4.3 Benefits for individuals and colleges

Multiple-choice questions with more than one correct answer in the questionnaire were used to investigate the opinions of referees, team leaders, participating students, and volunteers regarding the competition that affected their personal abilities and the school’s teaching activities and policies (Table 5).

**Table 5:** Personal abilities and schools’ teaching areas that could be affected by the Guanghua Cup

	Item	referees (%)	team leaders (%)	participating students (%)	volunteers (%)	*p* value
Personal abilities	Professional knowledge*	10 (31.25 %)	20 (66.67 %)	122 (73.50 %)	52 (47.28 %)	0.000
	Clinical skills*	8 (25.00 %)	18 (60.00 %)	139 (83.73 %)	30 (27.27 %)	0.000
	Self-recognition*	6 (18.75 %)	10 (33.33 %)	89 (53.61 %)	22 (21.00 %)	0.000
	Professional interest*	6 (18.75 %)	9 (30.00 %)	80 (48.19 %)	38 (34.55 %)	0.004
	Cooperation*	18 (56.25 %)	28 (93.33 %)	148 (89.16 %)	98 (89.09 %)	0.000
	Communication*	14 (43.75 %)	23 (76.67 %)	124 (74.70 %)	84 (76.36 %)	0.004
	Teaching methods	26 (81.25 %)	23 (76.67 %)	121 (72.89 %)	83 (75.45 %)	0.812
	Crisis management*	6 (18.75 %)	15 (50.00 %)	74 (44.58 %)	30 (27.27 %)	0.001
	Others*	2 (6.25 %)	0 (0.00 %)	1 (0.60 %)	0 (0.00 %)	0.046
Schools’ teaching	Improving the standardization of preclinical and clinical teaching*	26 (81.25 %)	28 (93.33 %)	157 (94.58 %)	73 (66.36 %)	0.000
	Updating teaching methods*	20 (62.50 %)	19 (63.33 %)	78 (46.99 %)	37 (33.64 %)	0.003
	Strengthening competitive consciousness	16 (50.00 %)	16 (53.33 %)	89 (53.61 %)	54 (49.09 %)	0.895
	Promoting communication and cooperation*	30 (93.75 %)	28 (93.33 %)	116 (69.88 %)	88 (80.00 %)	0.001
	Enriching experience in holding competitions*	12 (37.50 %)	20 (66.67 %)	64 (38.55 %)	38 (34.55 %)	0.017
	Encouraging education reform*	18 (56.25 %)	18 (60.00 %)	78 (46.99 %)	30 (27.27 %)	0.000
	Others*	2 (6.25 %)	0 (0.00 %)	0 (0.00 %)	0 (0.00 %)	0.029

*Represent statistically significant difference among four interview groups. *(*p* < 0.05)

In terms of improving personal ability, 81.25 % of referees pointed out that the competition promoted the “teachers’ teaching method”. There was no significant difference in the degree of agreement among all respondents on this question (*p*>0.05). The majority of team leaders (93.33 %), participating students (89.16 %), and volunteers (89.09 %) agreed that the competition enhanced cooperation.

Regarding the influence of competition on teaching activities and policies, the “competition could improve the standardization of preclinical and clinical teaching” was agreed with by 93.33 % of the team leaders and 94.58 % of the participating students. The majority of the referees (93.75 %), team leaders (93.33 %), and volunteers (80.00 %) declared that the competition promoted communication and cooperation.

#### 4.4 Suggestions for the future competitions

There were also multiple-choice questions in the questionnaire regarding advice about any items that should be added to future competitions (Table 6). The interviewees’ suggestions included caries removal and cavity preparation, cavity filling, three-dimensional (3D) digital scanning, and impression, with support rates ranging from 42.98 to 66.67 %. The suggestions for 3D digital scanning and impression accounted for the largest portion among referees and team leaders, with rates of 68.75 and 73.33 %, respectively, while the suggestion rate for students was 57.23 %, which was not significantly different among all the interviewees (*p* > 0.05). In addition, 73.49 % of the students recommended that composite resin filling should be added to the competition, which accounted for the majority of the student proposals. Other suggestions had a total proportion of 14.03 %, including oral health instructions, clinical case analysis, tooth extraction, root canal preparation, root canal filling, microscope-assisted precision dentistry, etc.

**Table 6:** Suggestions of other clinical skills that could be added to help make future competitions better

Item	referees (%)	team leaders (%)	participating students (%)	Total (%)	*p* value
Caries removal and cavity preparation	16 (50.00 %)	12 (40.00 %)	70 (42.17 %)	98 (42.98 %)	0.663
Composite resin filling *	14 (43.75 %)	16 (53.33 %)	122 (73.49 %)	152 (66.67 %)	0.001
3D digital scanning and impression	22 (68.75 %)	22 (73.33 %)	95 (57.23 %)	139 (60.96 %)	0.164

*Represent statistically significant difference among three interview groups. *(*p* < 0.05)

## Discussion

As a national clinical skills competition for dental undergraduate students in China, the competition enjoyed the widest coverage by involving dental schools from all different geographical regions of China. Compared with the China National Clinical Skills Competition for medical students [10], the Guanghua Cup was on a smaller scale and did not fully cover all oral dentistry-related knowledge and skills. However, the competition gradually increased people’s attention to undergraduate dental education, as shown by the increasing number of participating schools and visiting schools during the three years of the event. Compared with the 2nd and 3rd Guanghua Cups, fewer schools and competition items were involved in the 1st Cup. Therefore, we reported on and analyzed the 2nd and 3rd Cups, which may shed some light on modern dental education and provide referential significance for dental education policymakers, dental schools, teachers, and students.

### 1. The positive effects of the competition

According to the questionnaire data, collaboration skills were regarded as the most significant outcome of enrolling in the competition. Over the competition period, the majority of the required clinical skills could not be performed independently. For instance, rubber dam placement and periodontal and surgical sutures require well-coordinated assistants while the operator is working. In the process of mutual collaboration, students discussed and formulated their most appropriate and effective strategy. Since the deficiency of team-based learning is a common problem in China [11], teamwork and collaboration skills could be enhanced effectively through participation in such competitions, which could better promote students’ practical skills in the future [12, 13].

Compared to the 2nd Cup, the mean level of the participating students was enhanced in the stations related to periodontology, prosthodontics, and dental anatomy in the 3rd Cup (Table 3). The possible reasons for this are analyzed as followed. First, as the objective evaluation of each item of the competition was similar for each year, the students may have been better prepared as they knew the standard. Second, the schools paid more attention to enhancing the associated operative skills after being made aware of their deficiencies in the 2nd competition. Finally, the fact that the teachers applied the equipment (Real-time Dental Training and Evaluation System) that was introduced in the 2nd Cup to their daily teaching meant that the participating students did not perform nervously and anxiously on the related tasks. During their practice of clinical skills and preparation for professional knowledge, the students revised and consolidated their speculative learning, i.e., practice-based learning, which may have contributed to a solid base of theoretical knowledge in the participating students. Foreign colleges markedly attracted students and dentists to participate in the 3rd competition and assisted their students with a platform through which to interact and share ideas and eventually boost their English capacity [7].

According to our survey, all the participants were dental students in their fifth-year clinical practice stage who had finished all their theoretical and preclinical courses. Meanwhile, most schools utilized nonworking time for precompetition training to reduce the impact on the students’ clinical practice. Therefore, the competition schedule was unlikely to obstruct the student’s normal study schedule or curriculum learning by preparation before the competition. As one of the teaching purposes at the clinical practice stage is to improve one’s clinical operational skills, the contents of our competition that were designed to simulate the operation for patient treatment aimed to help students consolidate their clinical skills. Moreover, most dental students will attend the National Medical Licensing Examination after their graduation, and the contents of this examination are close to our competition items. Therefore, it can be deduced that participating in the Guanghua Cup is conducive for dental students passing this examination.

It is noteworthy that the competition aimed to test not only students but also teachers. First, participating colleges prepared for the competition following the teaching standards and methods required and advocated by the organizer, in which teachers regulated their clinical and teaching skills and improved their quality of education [14]. For instance, tooth carving and tooth molding teaching plan deficiencies were identified in the 1st and 2nd competitions. Therefore, some schools strengthened their training programs before these competitions. Thus, the performance of these programs in the 3rd competition significantly improved. For the other competitions, it was possible to adjust the programs’ teaching plans or increase the number of learning items to ensure that senior students had a firm grasp of dental anatomy. Second, the referees’ comments on students’ performance reflected daily teaching achievements and weaknesses, which might be significant for optimizing the faculty’s teaching methods. For example, the posture of dental operation and the consciousness of patient-friendly and aseptic techniques, which were not included in the scoring system, were repeatedly emphasized in the referees’ comments. Third, the competition provided an effective communication platform for different teachers to explore a wide range of learning opportunities [15].

In recent years, with the national economy’s high-speed development, the living standards in different regions have been elevated homogeneously. However, due to China’s large area, the education level of each geographical region is not balanced. During enrollment in the Guanghua Cup and other similar competitions, excellent dental schools from each geographical region of China were invited. Colleges and teachers were offered an opportunity to share their teaching concepts and experiences. As an influential national competition, the Guanghua Cup attracted participants’ attention throughout China and strengthened the participants’ communication.

Since the 2nd competition, digital objective evaluation systems (i.e., AccuDent and Ai Zhixing) have achieved significant outcomes for the evaluation of the two competition sections. These digital objective evaluation systems or equipment have provided quantitative evaluation data, which have not only reduced teachers’ subjective evaluation bias but also assisted students with important learning opportunities, thereby enabling them to critically self-assess themselves [15]. The application of medical clinical training simulators and digital evaluation systems in preclinical training and clinical skills competitions reflects the development trend of modern medical education [9, 16]. The digital evaluation model adopted in the Guanghua Cup provided significant ideas for participating universities related to the reform of clinical medical education.

### 2. Considerations for the future competitions

#### 2.1 How can we increase the overall quality of medical education and benefit more students?

First, providing a series of excellent works (no limit to competitional works), videos, and operational guidance is highly significant for instructing standardized clinical operations via a special website. Second, open registration could be adopted to promote the sharing of education resources (previous excellent works, teaching videos, operational guides, etc.). Even if only a few students are permitted to attend the final competition, other dental schools and students could benefit from the professional resources provided by the websites and the precompetition training or selections. Future goals will focus on evaluating the outcomes of these new measures.

#### 2.2 How can we ensure that the competition’s items and evaluation methods are close to those seen in clinical practice?

The proposals for future competitions were collected from all participants, including clinically relevant operative skills, such as composite resin filling, root canal treatment, and tooth extraction, as well as modern technologies, e.g., 3D digital intraoral scanning. In addition, interviewees also proposed case-based clinical simulation items to evaluate critical clinical thinking [17]. Future competitions can consider the addition of some of these items. Moreover, it is feasible to introduce dental simulation competitions, such as doctor-patient communication [18], to enhance students’ knowledge, skills, and professional attitudes; such competitions may ultimately enable students to apply such abilities to subsequent competitions. However, the issue of how to standardize the scoring to reflect fairness is a question that requires further scholarly attention.

#### 2.3 How can we come to more effective and convincing conclusions about the Guanghua Cup?

A limitation of our research was that we did not include control groups. To obtain more objective and impartial consequences in the future, we should simultaneously include nonparticipants as control groups. Furthermore, weare considering establishing more collaborations with the participating schools regarding comparing all the students’ (not limited to the participating students) professional performances before and after the competition, from which we would be able to determine whether the competition indeed plays a positive role in elevating students’ clinical skills and theoretical knowledge.

## Conclusions

In summary, the Guanghua Cup enjoyed the widest coverage by involving dental schools from all the different geographical regions of China. Dental students were able to demonstrate their skills in a competitive framework. The future development trend of such competitions will expand the number of competition items and attract more universities to participate in these competitions. At the same time, the evaluation criteria should be optimized.

## Data Availability

The datasets used and/or analyzed during the current study are available from the corresponding author on reasonable request.

## Abbreviations

The Guanghua Cup: The National Dental Undergraduate Clinical Skills Competition sponsored by Guanghua School of Stomatology, Sun Yat-sen University of China
SP: Standardized patient
CPR: Cardiopulmonary resuscitation
BP: Blood pressure
3D: Three-dimensional

## Declarations

### Authors’ information

Xiaolan Li, Ph.D., is an endodontist, university teacher, and director of undergraduate education at the Guanghua School of Stomatology, Sun Yat-sen University. Yanbo Shan is an undergraduate student at the Guanghua School of Stomatology, Sun Yat-sen University. She participated in the organization of the Guanghua Cup. Yangjingwen Liu, LLM, is a director of continuing education at the Guanghua School of Stomatology, Sun Yat-sen University. Yingwen Lin is an administrative staff member of the Education Affairs Department of the Guanghua School of Stomatology, Sun Yat-sen University. Lin Li is a director of student affairs at the Guanghua School of Stomatology, Sun Yat-sen University. Zhengmei Lin, Ph.D., is a professor, endodontist, university teacher, and vice dean in charge of stomatology education at the Guanghua School of Stomatology, Sun Yat-sen University. Shuheng Huang, Ph.D., is an endodontist, university teacher, and director of postgraduate education at the Guanghua School of Stomatology, Sun Yat-sen University. Yun Hong, Ph.D., is a university teacher and director of the Education Affairs Department at the Guanghua School of Stomatology, Sun Yat-sen University.

## References

[1] Zheng JW, Zhang SY, Yang C (2013). Current undergraduate and postgraduate dental education in China. J Dent Educ.

[2] Wu ZY, Zhang ZY, Jiang XQ (2010). Comparison of dental education and professional development between mainland China and North America. Eur J Dent Educ.

[3] Wang YH, Zhao Q, Tan Z (2017). Current differences in dental education between Chinese and Western models. Eur J Dent Educ.

[4] Sun H, Yang J, Kawashima N (2012). A brief comparison of curricula at dental schools in China and Japan. J Dent Educ.

[5] Wang SL, Li CY, Wang BK (2008). The educational standards ofthe stomatological of China. Chin J Stomatol.

[6] Li C, Zheng J, Guo C (2014). An introduction to clinical practice guideline for Chinese undergraduates in stomatology. Eur J Dent Educ.

[7] Wang X, Liu Y, Yang L (2020). The evaluation of stomatology English education in China based on ‘Guanghua cup’ international clinical skill exhibition activity. BMC Med Educ.

[8] Xu X, Xie Q, Zhou Y (2020). Effect of a standardized training with digital evaluation on the improvement of prosthodontic faculty’s performance in crown preparation: apre-post design. J Prosthodont.

[9] Tang L, Cao Y, Liu Z (2020). Improving the quality of preclinical simulation training for dental students using a new digital real-time evaluation system. Eur J Dent Educ.

[10] Jiang G, Chen H, Wang Q (2016). National Clinical Skills Competition: an effective simulation-based method to improve undergraduate medical education in China. Med Educ Online.

[11] Chen M, Ni C, Hu Y (2018). Meta-analysis on the effectiveness of team-based learning on medical education in China. BMC Med Educ.

[12] Der-Nigoghossian CA, Eldebs M, Karaoui LR (2015). Hints for successin ASHP’s clinical skills competition. Am J Health Syst Pharm.

[13] Cortez EJ, Boulger CT, Eastin T (2014). The Ultrasound Challenge 2.0: introducing interinstitutional competition in medical student ultrasound education. J Ultrasound Med.

[14] Zhou HJ, Wang H, Liang FX (2020). Review and thinking of fifth national clinical skill competition of acupuncture-moxibustion and tuina. Zhongguo Zhen Jiu.

[15] Lee C, Kobayashi H, Lee SR (2018). The role of digital 3D scanned models in dental students’ self-assessments in preclinical operative dentistry. J Dent Educ.

[16] Wolgin M, Grabowski S, Elhadad S (2018). Comparison of a prep check-supported self-assessment concept with conventional faculty supervision in a pre-clinical simulation environment. Eur J Dent Educ.

[17] Damon Dagnone J, Takhar A, Lacroix L (2012). The Simulation Olympics: a resuscitation-based simulation competition as an educational intervention. CJEM.

[18] Salerno N, Papanagnou D, Mahesh P (2018). Challenging hazards amidst observational simulation in the emergency department: advancing gamification in simulation education through a novel resident-led skills competition. Cureus.

